# Can improved agricultural technologies spur a green revolution in Africa? A multicountry analysis of seed and fertilizer delivery systems

**DOI:** 10.1111/agec.12533

**Published:** 2019-10-29

**Authors:** Joshua Ariga, Edward Mabaya, Michael Waithaka, Maria Wanzala-Mlobela

**Affiliations:** 1 Bill & Melinda Gates Foundation, Seattle, Washington, USA; 2 African Development Bank, Abidjan, Cote d’Ivoire; 3 The African Seed Access Index, Nairobi, Kenya; 4 African Fertilizer and Agribusiness Partnership, Johannesburg, South Africa

**Keywords:** Africa, Green Revolution, input markets, technology adoption, D24, O13, O55

## Abstract

Sub-Saharan Africa faces low agricultural productivity amid a confluence of trends that include rapid population growth, climate change, and the rise of the middle class. To raise productivity, governments—in partnership with donors and development organizations—have launched numerous initiatives to encourage the development of sustainable and competitive agricultural input markets. Despite these efforts, markets remain underdeveloped in most countries and access to affordable seeds and fertilizers remains a major challenge for smallholder farmers. This paper explores evidence from recent multicountry analyses of input delivery systems to assess the possibility of a Green Revolution in Africa. It describes use and adoption levels, challenges, policy and regulatory issues, and investments needed to expand smallholder access to these productivity-enhancing agricultural technologies.

## 1 Introduction

In contrast to North America and Europe, whose populations are projected to decline in the coming decades, Africa and Asia are expected to grow, with Africa’s population surpassing that of Asia by 2075 (United Nations, 2017). In sub-Saharan Africa (SSA), rapid population growth is putting pressure on land resources and demand for food. The region’s agricultural sector, which is mainly composed of smallholder farms, contributes 15% to total national gross domestic product and accounts for more than one-third of foreign exchange (EIU, 2012). Increased agricultural productivity is therefore crucial to sustainable and more inclusive economic growth in the region.

Most agricultural resources in SSA are allocated to cereal crops. Maize accounts for more than 15% of the estimated 200 million hectares (ha) of cultivable land, almost half of the calories and protein consumed in East and Southern Africa, and one-fifth of the calories and protein consumed in West Africa. Production and consumption of rice, wheat, and sorghum are also increasing steadily in SSA (McCauley, 2015). However, cereal yields are generally low and stagnant, at around 1 ton/ha compared to 4 tons/ha in other developing regions. This can be traced to a range of factors, including low adoption of improved agricultural technologies, limited irrigation infrastructure and extension services, and weak linkages to output markets. As a result, SSA is a net food importer and is vulnerable to global food price shocks. To achieve food security and economic growth, the region must significantly increase its agricultural production and productivity.

## 2 Problem Statement

To feed its rapidly growing population, SSA must apply innovative approaches to raise agricultural productivity and incomes. The region accounts for more than 10% of the world’s population but less than 1% of global fertilizer use; fertilizer use is only 15 kg/ha, compared to the world average of 124 kg/ha (The Economist, 2016). Soil nutrient depletion rates are severe, exceeding 60 kg/ha (Wanzala & Groot, 2013). Adoption of improved seeds among smallholder farmers varies widely across crops and countries but is low overall.

Agricultural input systems must become more competitive and sustainable so that farmers can access quality fertilizers and improved seeds at the right time and place and use recommended rates. In recognition of this imperative, African governments and regional economic communities, together with donors and development organizations, have launched numerous initiatives to develop sustainable agricultural input systems (AU, 2014; Minot & Benson, 2009; NEPAD, 2011). These include efforts to encourage private-sector participation and investment in input supply chains.

This paper assesses whether the technologies embodied in improved seeds and chemical fertilizers can stimulate a Green Revolution in Africa. The paper has three interrelated objectives: to describe the status and key features of seed and fertilizer systems in SSA; to distill some key lessons about these markets from the literature; and to discuss related policy implications and ways that governments and industry can help improve smallholder farmers’ access to and use of productivity-enhancing inputs.

## 3 The Seed System

### 3.1 Improved seeds

Access to high-quality, locally adapted, genetically improved and affordable seeds is essential to boosting agricultural productivity (Zeng et al., 2015). Adoption of improved seeds can enhance productivity by increasing nutrient use efficiency and improving immunity to biotic and abiotic stresses, resulting in higher incomes for farmers (Bezu, Kassie, Shiferaw, & Ricker-Gilbert, 2014). Moreover, gains from improved varieties can extend off-farm to benefit consumers and other actors along the value chain (Evenson & Gollin, 2003).

Seed systems in most SSA countries are still relatively underdeveloped, and many farmers plant open-pollinated varieties from previous harvests (McGuire & Sperling, 2013). Most farmers have yet to take the advantage of new crop varieties developed by their country’s National Agricultural Research System or International Agricultural Research Centers, mainly due to weak seed production and distribution linkages, limited availability, lack of knowledge, cost, risk aversion, and preference for landrace varieties (Mabaya, Omanga, & DeVries, 2013).

### 3.2 Adoption of improved varieties

Based on the status of breeding and variety release, the policy and regulatory environment, private-sector participation, and the effectiveness of distribution systems, the seed industry in most SSA countries is in the emerging, early growth, or growth stage, as shown in Table 1. Adoption of improved or modern seed varieties in SSA varies widely by location and crop. Moreover, the definitions of *improved seed* and *adoption* vary among studies, often resulting in vastly different estimates even for the same country, crop, and year (Glover, Sumberg, & Andersson, 2016; Walker et al., 2014). In this paper, the adoption rate of improved seed for a crop is defined as the percentage of total land under cultivation that is planted with either quality declared or certified seed.

**TABLE 1:** Stages of growth of the seed industry in SSA

	Stage 1 Nascent	Stage 2 Emerging	Stage 3 Early growth	Stage 4 Growth	Stage 5 Mature
Improved seed adoption	Aid/relief programs Few commercial farmers	<2.5% Innovators	2.5-16% Early adopters	16-84% Critical mass	>84% All but the latest adopters
Breeding and variety release	No original breeding No formal variety release process	Some original breeding Variety release formalized	Strong breeding systems	Robust breeding pipeline Favorable seed policies	Mostly driven by the private sector
Policy and regulation	Nonexistent in most cases	Basic and incomplete	Evolving seed policy and regulations	Policies and regulations established and enforced	Industry driven and self-regulating
Private-sector participation	No private seed companies	Few small seed companies	Many small/medium seed companies	Many stable seed companies	Mostly large seed companies
Distribution system	Imported seed only	Limited agro-dealer network	Growing agro-dealer network	Strong agro-dealer network plus specialized outlets	Vertical integration
Country examples	South Sudan, Liberia, Sierra Leone, Angola, and DRC	Niger, Mali, Senegal, Madagascar, and Ivory Coast	Burkina Faso, Ghana, Ethiopia, Tanzania, and Nigeria	Uganda, Zambia, Kenya, Malawi, and Zimbabwe	South Africa

*Source:* Adapted from Mabaya et al., 2013.

Over the past two decades, the area planted with modern varieties of maize, including both hybrids and open-pollinated varieties, has increased significantly. In 2006, it was 33% of the area in Eastern Africa and 38% in Southern Africa, excluding South Africa (Mason, Jayne, Chapoto, & Donovan, 2011); in 2005, it was 15% in Western Africa (Alene et al., 2009). In the early 2000s, adoption rates reached 60% for modern varieties of wheat and 40–50% for rice (Evenson & Gollin, 2003).

The situation for maize is different from that of other crops, with adoption and yearly purchase of seeds increasing following major initiatives to foster market-led technology adoption (Toenniessen, Adesina, & DeVries, 2008). Examples of this trend include an emerging commercial maize seed sector in Kenya, public maize dissemination in Ethiopia, a strong association between nongovernmental organizations (NGOs) and private companies in seed marketing in Ghana, and publicly subsidized input programs in Malawi and Zambia (Scoones & Thompson, 2011). In several countries, including Malawi and Zambia, the maize model is embedded in national subsidized input programs that target national food security and enterprise development. However, in both Malawi and Zambia, these programs use a major share of the government budget for agriculture (Chinsanga, 2011; Nakaponda, 2011), which indicates limited sustainability. Smale, Byerlee, and Jayne (2011) and Scoones and Thompson (2011) question whether the maize model is economically viable and institutionally sustainable and suggest that it is not applicable to seed systems for other food crops.

### 3.3 Structure and development of formal seed systems

The formal seed system is “a deliberately constructed system, which includes a chain of activities leading to clear products: certified seeds of verified varieties” (Sperling & Cooper, 2003). Formal seed systems in SSA are highly fragmented and complex. In developed countries where formal seed systems are well established, the entire seed value chain from research through distribution is often controlled by one or two private companies that have vertically integrated over the years through mergers and acquisitions. In contrast, Africa’s seed sector involves numerous players, sometimes with conflicting interests, operating in a loosely integrated value chain. Within the formal seed sector, two models are common in SSA. One is the public/parastatal model, in which a state agency multiplies and processes seed that is often protected from competition by statutory instruments. The other is the private-sector model, in which seed production, processing, and marketing are done mainly by private enterprises. Under both models, seed is mostly marketed and distributed through networks of rural agro-dealers or NGOs that distribute seeds through aid programs. The structure of the formal seed sector is constantly changing to cope with the dynamic macroenvironment, which includes seed policy and regulations, agro-ecological conditions, donor initiatives and investments, advocacy and special interest groups, and socioeconomic factors.

As shown in Table 1 and described further below, formal seed sectors in SSA countries are at different stages of development. Note that the table is somewhat biased toward maize, which accounts for the bulk of formal seed sector sales. Maize often leads development of the seed sector, followed closely by other grains, while pulses and vegetatively propagated crops lag behind. The five stages of seed sector development are:

- **Stage 1 – Nascent**: Many African countries are still in the nascent or embryonic stage of seed sector development, in which key policy and institutional frameworks for a formal seed sector are absent. The little seed that is available is imported and used almost exclusively by commercial farmers or relief programs. Countries in this category include South Sudan, Liberia, Sierra Leone, Angola, and the Democratic Republic of the Congo (DRC).
- **Stage 2 – Emerging**: Countries with emerging seed sectors often have some original breeding programs and a formalized variety release process supported by a basic policy and regulatory framework. Seed production and distribution are conducted by a handful of seed companies and/or government parastatals. Adoption of improved seed in these countries is limited to innovating farmers served by NGOs and a limited agro-dealer network. Countries with an emerging seed sector include Niger, Mozambique, Rwanda, Mali, Senegal, Botswana, Madagascar, and Côte d’Ivoire.
- **Stage 3 – Early growth**: Countries with well-established breeding programs and evolving seed policies are in the early growth stage. Startup seed companies produce and sell a limited range of staple crops to early adopting farmers. Countries in the early growth stage include Burkina Faso, Ghana, Ethiopia, Tanzania, and Nigeria. Both governments and NGOs are still significant players in this stage, supported by a growing agro-dealer network.
- **Stage 4 – Late growth**: Spurred by private companies, countries in the late growth stage have well-established seed sectors supported by strong breeding programs and seed policies that support private-sector participation. In this stage, private-sector activity is highly competitive, often with multinational and domestic seed companies producing a wide array of high-quality seeds distributed through a strong agro-dealer network plus specialized outlets. Only a handful of Eastern and Southern African countries are in this stage: Uganda, Zambia, Kenya, Malawi, and Zimbabwe.
- **Stage 5 – Mature**: The most advanced stage of seed sector development is characterized by a self-regulating and fully privatized seed sector that is on par with that of developed countries. Due to mergers and acquisitions, the number of seed companies is lower than in the growth phases. Most participating companies are vertically integrated, with in-house breeding programs and a tightly managed distribution system. The role of the government is minimal and mostly in line with private-sector needs. In SSA, only South Africa has reached the mature stage.

### 3.4 Ten key insights about the formal seed sector in SSA

This section summarizes emerging trends in the formal seed sector across the continent, based on 13 comprehensive studies covered by The African Seed Access Index (TASAI). TASAI measures, tracks, and compares enabling environments across SSA countries. For the top four grain and legume crops in each country, the index tracks 20 indicators in five categories: Research and Development, Industry Competitiveness, Seed Policy and Regulations, Institutional Support, and Service to Smallholder Farmers. In 2016 and 2017, TASAI conducted studies in the DRC, Ethiopia, Ghana, Kenya, Madagascar, Malawi, Mozambique, Senegal, South Africa, Tanzania, Uganda, Zambia, and Zimbabwe.

**FIGURE 1:**
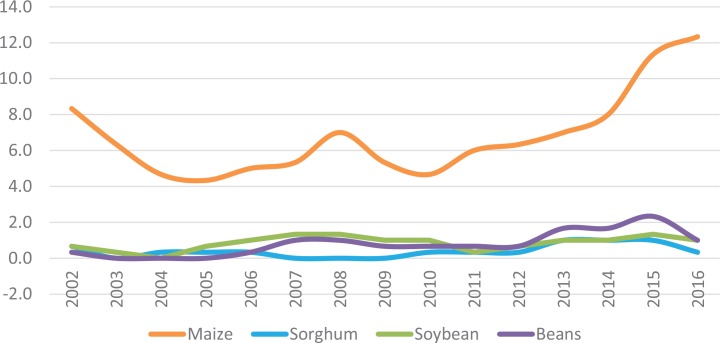
Zimbabwe’s 3-year moving average of varieties released (2000–2016) [Color figure can be viewed at wileyonlinelibrary.com] *Source:* Mabaya et al., 2017.

Ten key insights emerged from our review of the 13 country studies:

1. **Maize dominates crop breeding programs.** In all but three of the countries, maize dominates formal breeding programs in both research and development investments and outputs. Figure 1 shows the 3-year moving average of varieties released in Zimbabwe from 2000 to 2016, including an average of about eight new maize varieties per year. Maize accounts for 67% of the new varieties across all 13 countries tracked by TASAI. In most of the countries, maize breeders account for at least half of the active breeders for the top four food crops, and maize dominates the variety release catalog, with at least two-thirds of the varieties released in the past 3 years. In extreme cases, such as Ghana and Malawi, maize is the only crop among the top four food crops with varieties released since 2013.
2. **Old varieties persist despite the introduction of new varieties.** In all 13 countries, many new and better-performing varieties have been released in the past decade. Most of the new varieties have new traits, such as improved nutrition, and climate-smart features, such as drought tolerance, frost resistance, and early maturity. However, in most countries, the average age of some crop varieties on the market is more than 15 years. Examples include sorghum and cowpeas in Kenya, maize and groundnut in Madagascar, groundnut in Malawi, beans in Tanzania, and all crops in Senegal. Without policies to retire dated varieties, popular old varieties persist.
3. **Local private seed companies play an important role.** Apart from South Africa, Zambia, and Zimbabwe, at least three-quarters of the active seed companies in most countries are local. However, regional multinationals are expanding their presence through strategic partnerships, subsidiaries, and acquisitions.
4. **In mature seed sectors, the seed industry is consolidating.** Between 2014 and 2016, industry consolidation was the trend in South Africa and Zimbabwe. During this period, both countries witnessed several mergers and acquisitions in the seed industry, which could result in reduced competition in the future.
5. **The government role in seed production is diminishing, with exceptions.** Government-owned seed companies still operate in Ethiopia, Kenya, Madagascar, Tanzania, and Zimbabwe. However, except for Ethiopia and Kenya, the market share per crop of current and former government parastatals is less than 10% in most cases.
6. **Regional seed import and export processes have notably improved.** By volume, maize is the most traded seed crop in SSA. Kenya, South Africa, Zambia, and Zimbabwe are net exporters of maize seed. For most countries, the seed import/export process takes less than 30 days on average. In Tanzania, Uganda, and Zambia, the process can take less than 10 days. However, challenges remain with the seed import process in Ethiopia and Ghana and with the export process in Zimbabwe.
7. **Policy instruments are good, but their implementation is weak.** Most seed companies expressed a high level of satisfaction with the quality of seed policy instruments, including the seed policy, seed act, seed regulations, and seed strategy in their country. However, most of them expressed a high level of dissatisfaction with the level of enforcement and implementation of these instruments. This is especially true for Ethiopia, Ghana, Madagascar, and Malawi. In the worst cases, such as in Senegal, weak enforcement at all stages of the seed value chain is leading to poor-quality seeds on the market.
8. **Steady efforts toward privatization of seed inspection services are underway.** South Africa, Zambia, and Zimbabwe are the only countries tracked by TASAI that have more private than public seed inspectors. This has translated to greater efficiency in seed inspection services and high satisfaction rates among seed companies. Over the past few years, Ghana, Kenya, Malawi, Mozambique, Tanzania, and Uganda have taken substantive steps in the training and accrediting of independent seed inspectors to complement current government efforts.
9. **The challenge of fake seed persists.** Fake seed continues to be a significant challenge in all of the countries except South Africa. Seed companies in Madagascar, Malawi, Senegal, Zambia, and Zimbabwe reported more than 20 cases of fake seed in 2016. This is likely an underestimate because most cases of fake seed are not reported. In Ghana, Madagascar, Malawi, and Uganda, seed companies are dissatisfied with government efforts to address the problem of fake seed, but several countries are taking notable steps to address the challenge. The Seed Trade Association of Kenya (STAK), working closely with the government seed regulator, is leading the effort to have labels inserted on all packets of seed that can be authenticated by SMS.
10. **Seed trade associations are an important link between industry and government.** Seed trade associations provide a wide range of services to the seed industry and its members, ranging from advocacy to promotional activities. The South African National Seed Organization is in many respects is a model seed trade association; its members rate the association’s performance as excellent across key service areas. Other associations, such as STAK, Seed Trade Association of Malawi, Zambia Seed Trade Association, Tanzania Seed Trade Association, and Uganda Seed Trade Association, are well regarded and have a good working relationship with their governments in addressing critical issues affecting the industry. Ethiopian Seed Association, Malagasy Association for Seed and Plant Professionals, and National Seed Trade Association of Ghana are relatively young and are growing quickly. The Association for Promotion of the Seed Sector in Mozambique and Senegal’s National Union of Seed Professionals are not very active, resulting in poor representation of the seed industry to their governments and other stakeholders.

## 4 The Fertilizer System

### 4.1 Inorganic fertilizers

According to Norman Borlaug, who is known as the father of the Green Revolution in Asia and Latin America, seeds are the “catalysts that ignited the Green Revolution” and fertilizers are “the fuel that has powered its forward thrust” (Carter, Laajaj, & Yang, 2014). At least 30–50% of global crop yield is attributable to the use of inorganic fertilizers (Stewart, Dibb, Johnston, & Smyth, 2005; Stewart & Roberts, 2011). Despite the significance of fertilizers for food security, the majority of SSA farmers use little or no chemical fertilizer. This is partly due to limited access to financing and underdeveloped fertilizer delivery systems that are characterized by high transaction costs.

### 4.2 Fertilizer supply

Africa is well endowed with natural resources, oil, phosphates, and gas for use in fertilizer production (Van Kauwenbergh, 2006, 2010), particularly in Morocco, which is a significant player in the global phosphate market (Wanzala & Groot, 2013). Nevertheless, SSA is a net importer of fertilizer with some countries being minor producers, while others have plans to invest in manufacturing and blending. OCP of Morocco is currently expanding its marketing, manufacturing, and blending operations in SSA, working closely or in partnership with governments (Makepeace, 2017)—including the government of Nigeria, which has the capacity to produce 500,000 tons of nitrogen fertilizer per year. South Africa has four local manufacturers whose combined output in 2016 was 1.12 million tons, or 56% of total fertilizer output in the country, while Kenya and Ethiopia have a few blending facilities.

### 4.3 Fertilizer consumption

Excluding South Africa, SSA is the world’s fastest growing fertilizer market, averaging 8% growth per year since 2008. In 2012 and 2013, the market was estimated at 3.2 million tons (MT) of nutrients, with nitrogen, phosphorous, and potash accounting for 1.8, 0.9, and 0.5 MT respectively, which is less than 2% of the total world consumption. As a result of the focus on food security and subsidies, approximately 40% of the estimated 4.7 MT of fertilizer nutrients consumed in 2017 was used on maize (Heffer & Prud’homme, 2018).

**FIGURE 2:**
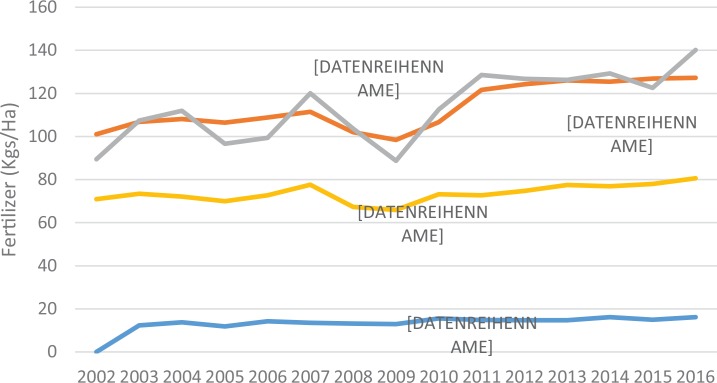
Fertilizer use by region (kg/ha of arable land) [Color figure can be viewed at wileyonlinelibrary.com] *Source:* World Bank database.

Despite this growth, fertilizer use is still far below the average in other developing regions, as illustrated in Figure 2. Although use varies across countries, average application rates have increased from approximately 8 kg in 2009 and 13 kg in 2013 to the current level of approximately 15 kg of nutrients per ha of cultivated land (Heffer & Prud’homme, 2018; Figure 2). This is substantially lower than the targeted rate of 50 kg of product per ha in the 2006 Abuja Declaration, which is equivalent to approximately 23 kg of nutrients per ha for urea fertilizer (NEPAD, 2011). The low application rates have led to increased nutrient mining in SSA, which was estimated at 60 kg/ha during the mid-1990s (Henao & Baanante, 1999). Total fertilizer consumption in Africa will need to increase substantially to meet the agricultural growth targets set in national agricultural development plans (IFDC, 2015). For example, Uganda would have to increase fertilizer consumption sixfold—from an estimated 45,000 MT in 2014 to approximately 306,000 MT—to meet the country’s agricultural growth targets (Table 2).^1^

**TABLE 2:** USAID Feed the Future Initiative fertilizer assessments: estimated quantities (‵000 MT)

	Tanzania	Zambia	Kenya	Ethiopia	Ghana	Mozambique	Uganda	Rwanda
Year	2012	2013	2012	2012	2012	2012	2014	2014
Current use	263	250	489	500	200	50	45	35
Additional needed to meet production targets	265	250	421	700	370	250	261	109
Total fertilizer required	528	500	910	1,200	570	300	306	144
Increase over current	2.0x	2.0x	1.9x	2.0x	2.7x	6.0x	6.5x	4.1x

*Source:* IFDC, 2015.

### 4.4 Fertilizer response rates

A major factor affecting demand for fertilizer is the *response rate*, which is measured as the value of the additional quantity of output that is attributable to one more unit of input compared to its cost, also known as the *value-cost ratio* (VCR). Some examples are provided in Table 3, which estimates the potential contribution of fertilizer to transforming the agricultural sector in SSA.

**TABLE 3:** Recent estimates of fertilizer application and response rates in SSA

Source	Geographic focus	% Maize fields receiving commercial fertilizer use	Application rate among users	Estimated average (AP) or marginal (MP) crop response to nitrogen (N) (kgs crop/kg N)	Estimated average (AVCR) or marginal value-cost ratio (MVCR)
Sheahan, Black, and Jayne (2013)	20 districts of Kenya where maize is commonly grown; 5 years of data (1997–2010)	64% (1997)–83% (2007)	26 kg N/ha (1997)–40 kg N/ha (2010)	AP: 21 kg maize/kg N MP: 17 kg maize/kg N	AVCR: from 1.3 (high-potential maize zone) to 3.7 (eastern lowlands)
Morris, Kelly, Kopicki, and Byerlee (2007)	W/E/S Africa			E/S Africa: 14 kg maize/kg N (median) W. Africa: 10 kg maize/kg N (median)	AVCR: E/S Africa: 2.8 W. Africa: 2.8
Minten, Koru, and Stifel (2013)	Northwestern Ethiopia	69.1% of maize plots fertilized	65.3 kg N/ha	MP = 12 kg maize/kg N on-time planting; 11 kg maize/kg N late planting	1.4–1.0 (varying by degree of remoteness)
Burke, Jayne, and Black (2017)	Zambia (nationally representative); 2001, 2004, and 2008 36–38% of maize fields; 45–50% of maize area	35.2 N/ha maize	9.6 kg maize/kg N	0.3–1.2 (depending on soil pH level for 98% of sample)
Ricker-Gilbert and Jayne (2012)	Malawi (national panel data)	59% of maize fields	47.1 N/ha maize	8.1 kg maize/kg N	0.6–1.6
Liverpool-Tasie, Omonona, Sanou, and Ogunleye (2017)	Nigeria (national LSMS survey data)			8.0 kg maize/kg N 8.8 kg rice/kg N	*>*2.0 *>*2.0
Mather et al. (2016)	Tanzania (national LSMS survey data)	15.9% (2009) 20.6% (2011) 17.9% (2013) 55.6 N/ha maize	7.8 kg maize/kg N (highlands) 5.7 kg maize	MVCR 0.94–1.23 (varies by year) MVCR 0.71–1.08

*Source*: Adapted from Jayne and Rashid (2013) and sources listed in the table.

**FIGURE 3:**
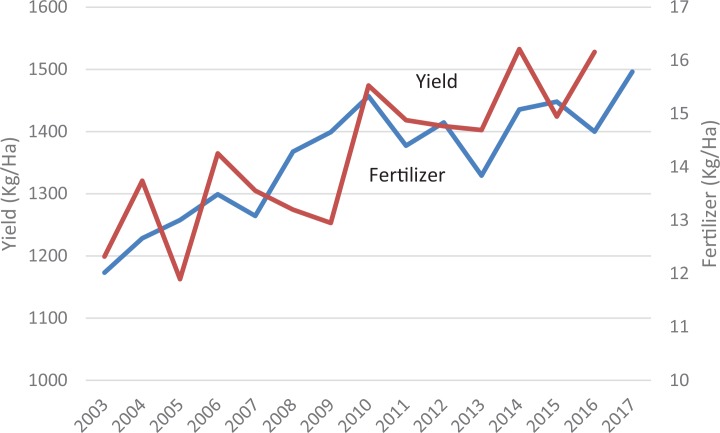
Cereal yields and fertilizer application in SSA (kg/ha of arable land) [Color figure can be viewed at wileyonlinelibrary.com] *Source:* World Bank database.

**FIGURE 4:**
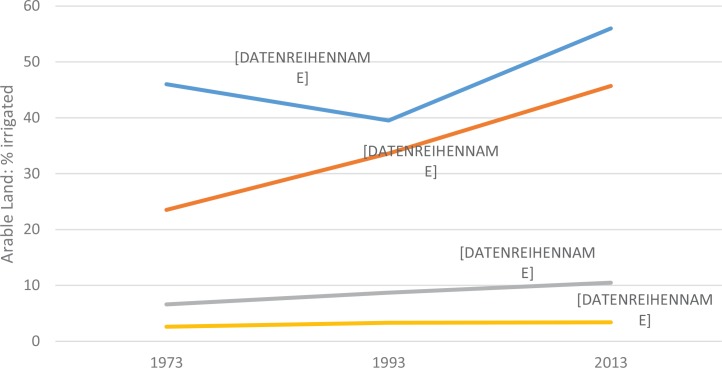
Irrigated land in various regions of the world (% of arable land) [Color figure can be viewed at wileyonlinelibrary.com] *Source:* UN FAO, electronic files, and website.

Figure 3 depicts fertilizer application and average cereal yields in SSA over the period 2000–2015. It is important to note that yield levels also depend on seeds, management, and soil organic matter. A VCR of 2 is typically the benchmark for the profitability of fertilizer use (Sauer & Tchale, 2009; Xu, Guan, Jayne, & Black, 2009). Few of the VCR estimates in Table 3 exceed 2, with most falling between 1 and 2. Thus, most survey-based studies suggest marginal or moderate levels of fertilizer profitability, and only when risk and other unmeasured costs are not considered. One reason for this low VCR, or fertilizer profitability, is that water for irrigation— mainly from rainfall—is limited. Figure 4 shows that less than 5% of arable land in SSA is irrigated, and less than 10% is projected to be irrigated by 2060 (You et al., 2012). A second reason for low fertilizer profitability is that poor soil quality or structure, with low organic matter and high acidity, significantly limits the efficacy of fertilizer. Third, heavy population pressure has reduced or eliminated fallow periods, leaving insufficient time for the land to recuperate. And finally, years of monocropping culture encourages mining of certain nutrients. These four factors have a bearing on recommendations regarding the use of fertilizers; unless these constraints are tackled holistically, efforts to improve smallholder adoption of fertilizers will face difficulties.

### 4.5 Fertilizer markets and delivery systems

Based on the available literature, fertilizer markets in SSA can be placed in four categories, ranging from nascent to developed, as shown in Table 4 (Ariga & Jayne, 2009; Jayne, Mason, Burke, & Ariga, 2018). The nascent category has fertilizer use at very low levels, mostly using free supplies provided by NGOs to stimulate demand. Emerging markets have less than 5% of farm households using inorganic fertilizers, mostly from NGO- and government-supported programs. Markets in the early growth phase have private-sector participation and a growing agro-dealer network but an underdeveloped policy and regulatory system and heavy state intervention. Developed fertilizer markets, like those in South Africa and possibly Kenya, have a relatively informed farmer base and are mostly led by the private sector and include blending and manufacturing activities.

**TABLE 4:** Stages of growth of fertilizer markets in SSA

	Stage 1 Nascent	Stage 2 Emerging	Stage 3 Early growth	Stage 4 Late growth
Adoption of inorganic fertilizers	Zero to low; mainly via aid/relief programs; few commercial farmers	*<*5% households in scattered locations	15–20% households use chemical fertilizers	*>*20%
Fertilizer blend-ing/manufacturing	None	1–2 blending plants	*>*2 blending plants	Blending and manufacturing plants
Policy and regulation	Regulatory system is state focused; no fertilizer policy	Limited; mainly reliant on decrees or command system from the state	Fertilizer policy and regulatory framework exist but are outdated	Fertilizer policy and regulatory framework is up to date
Private-sector participation	State-run market	Few private players, mostly NGOs/donors; poor infrastructure and information	Mainly state managed, with some private-sector players; heavy fertilizer subsidies	Mostly private sector; fewer subsidies, more reliable policy environment
Distribution system	Procurement and distribution managed by the state	Few importers, thin agro-dealer network	Growing agro-dealer network	Strong agro-dealer network (owned by importers and independent)
Country examples	South Sudan, Liberia, Sierra Leone, Angola, and DRC	Niger, Mozambique, Mali, Senegal, Madagascar, and Ivory Coast	Burkina Faso, Ghana, Ethiopia, Tanzania, and Nigeria	South Africa and Kenya

*Source*: Data collected by the authors.

**FIGURE 5:**
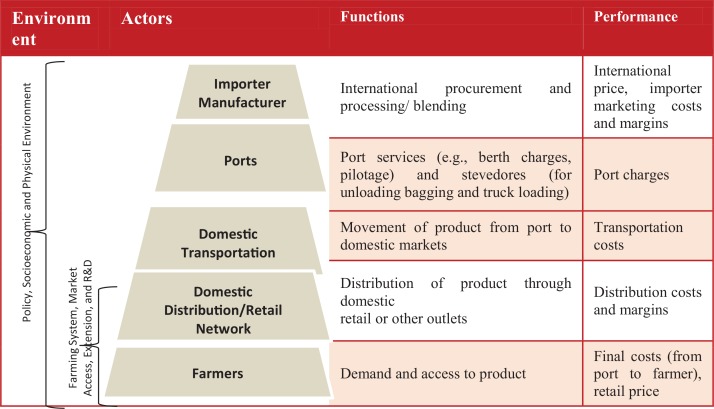
Typical structure of a fertilizer supply system in sub-Saharan Africa [Color figure can be viewed at wileyonlinelibrary.com] *Source*: Authors.

Apart from the level of market development, the structure of fertilizer delivery systems in SSA depends heavily on the availability of good infrastructure and financial services as well as enabling policies. A typical fertilizer supply chain in SSA starts with product from an international or local source that is shipped or transported by rail or road to the destination country or location. The product is unloaded and bagged at the receiving port and then transported inland to wholesale and retail stores. Most farmers access fertilizer from local agro-dealer stores; others, especially those buying in large quantities, purchase it directly from wholesalers and importers (Figure 5). Variants of this model exist, influenced by government procurement systems, the presence of subsidy programs, the size of private-sector investments, and other factors (Jayne et al., 2018).

### 4.6 Key challenges to improving delivery systems

Several studies (Gregory & Bumb, 2006; IFDC, 2007; Morris et al., 2007; Wanzala & Groot, 2013; Wanzala et al., 2009) have identified key constraints on smallholder farmers’ access to fertilizers. They can be categorized as supply constraints (related to procurement and manufacturing or production); demand constraints; and constraints in the enabling environment (related to policies and regulations, infrastructure, and financial services). Although a number of new fertilizer plants or plant renovations are in the pipeline, most notably in Nigeria, most fertilizer consumed in SSA is still imported. Most imports are of relatively small volume, and free-on-board prices and the costs of shipping, port clearing, and transportation tend to be high.

Factors contributing to weak demand for fertilizers include lack of information or poor information on agronomic and other extension services, limited access to soil-testing services, reliance on rudimentary technologies (such as hand-held hoes), use of fertilizers that do not fit specific soil and crop needs, long distances to the nearest agro-dealers, unreliable government-led delivery systems, risks associated with reliance on rainfall, and low yield response rates (Table 3).

Policy-related constraints include lack of clarity and consistency in fertilizer regulations and weak institutional frameworks, leading to fluid policies enacted in an ad hoc manner that depends on who is leading the ministry. State subsidies lead to risk and uncertainty in markets, particularly for the private sector, thereby reducing incentives and investment. Lack of regional harmonization stifles competition, cross-border trade, and the introduction of new products that could provide balanced nutrition and improve yield response rates.

Infrastructure challenges range from ill-equipped port facilities (leading to high port-clearing charges) to poor road and rail networks, which affect delivery times and hence the quality and cost of fertilizer at the farm gate. Financial constraints are experienced by actors at all levels of the value chain due to strict collateral requirements and the high cost of credit, with annual interest rates hovering around 30%.

### 4.7 Nine insights about fertilizer markets in SSA

Despite general agreement that fertilizer supply systems led by the private sector are more efficient than those led by the public sector (Jayne et al., 2018; Mather, Waized, Ndyetabula, Temu, & Minde, 2016), government subsidy programs and regulatory interventions continue to shape market dynamics. The following seven insights are distilled from the literature cited earlier:

1. **Fertilizer markets in SSA are complex.** Given broad diversity in the structure, operations, and performance zof fertilizer markets in Africa, it is helpful to sort the markets into “archetypes” that inform appropriate policy approaches across a number of countries. For nascent markets, the best way to increase access to fertilizers is through demonstrations and farm trials where farmers can see the results of using fertilizers and receive information. In some instances or locations, public support in the form of subsidies may be necessary. In more developed markets, such as in Kenya and South Africa, the best approach may be to foster a business environment that promotes private-sector expansion and facilitates registration of new products that target soil and crop nutrient needs. Subsidies in this case should be limited to selective assistance to poor households that have the capacity to sustain the momentum on their own within a predetermined time span (such as a few seasons).
2. **Global investors have renewed interest in the Africa fertilizer market.** Global fertilizer companies are increasingly interested in and investing in SSA fertilizer markets. This trend will have implications for the range and prices of products. A shift is already underway from commodity fertilizers to more balanced nutrition using compounds and blends.
3. **Fertilizer markets are evolving from centralized control to competitive, market-based systems.** Governments are gravitating toward policies and regulations that facilitate private-sector investments while still giving subsidies to vulnerable farmers. In this regard, research institutions and development partners can play a crucial role in providing accurate and current evidence-based analysis to help governments make appropriate policy decisions.
4. **Fake and adulterated fertilizers have a serious impact on fertilizer demand and crop yields.** Governments should establish regulatory and quality control systems that are adapted to local conditions but do not restrict trade.
5. **National and regional fertilizer trade and agro-dealer associations, where they exist, are weak.** Astheroleof the private sector in fertilizer markets expands, these associations should be strengthened so that the private sector can interact with government in a unified and effective manner and promote regional trade.
6. **The policy environment is a critical determinant of the structure and operations of agricultural input markets and therefore their performance.** Sheahan and Barrett (2017) found that, controlling for other factors, some combination of policy, institutional, and macroeconomic variables explains about 50% of the variation in input use. Key policy and regulatory challenges include subsidy programs, inadequate port and transport infrastructure, insufficient access to financing among fertilizer-sector players, weak regulatory systems, and the absence of mutual recognition of fertilizer standards and divergent regulatory requirements that stifle regional trade.
7. **Policy harmonization for fertilizers is weak and lags behind that for seeds.** The Economic Community of West African States (ECOWAS) has a regional fertilizer regulatory framework, but adoption at the country level has been slow. The Common Market for Eastern and Southern Africa and the East African Community (EAC) have initiated the adoption process, but it has been stalled for a number of years. No movement has taken place on this front in the Southern African Development Community (SADC). The regional harmonization process needs to be completed in EAC and SADC and domestication fast-tracked in ECOWAS to facilitate the growth and sustainability fertilizer markets in SSA.
8. **A holistic approach is required to raise crop productivity.** It is increasingly clear that raising yields will require interventions beyond promotion of fertilizers—including soil mapping and testing and an integrated soil fertility management approach that includes improved seeds. Fertilizer blending and the use of micronutrients are expanding and should be encouraged.
9. **Countries should invest in educating farmers about appropriate fertilizer use.** Sheahan and Barrett (2017) found that the integrated soil fertility management approach has not been adopted; some households use organic fertilizers without complementing it with inorganic fertilizers, and others use improved seed without accompanying inorganic fertilizer. Furthermore, farmers do not adjust input application rates to soil conditions and other factors.

## 5 Conclusions

The need to address challenges associated with input delivery systems in SSA is urgent, particularly in light of major regional trends that include rapid population growth, rising food demand, and increasing reliance on food imports. One key trend is the growth of the middle class, which will increase the demand for high-value foods, such as meats and vegetables, whose production requires substantial use of inputs and also has implications for the environment.

To meet these demands, policymakers and development partners must ensure enabling policy environments for private-sector investment. Moreover, regional member countries must urgently harmonize their regulatory systems and quality standards to encourage international trade and competition. Governments should limit their role to providing support in areas, such as extension services, research and development, policy formulation, and regulatory enforcement, as well as working with the private sector to eliminate fake inputs. Political will is necessary to tap into the large regional markets through harmonized regulations within and between regional economic blocs. Finally, both public- and private-sector players should take advantage of the growing information and communications technology environment in SSA to develop data and information systems that will strengthen markets, provide farmers with relevant agronomic and market information, and ensure the traceability of high-quality inputs. Building the capacity of both public and private entities in this domain and creating partnerships will reduce the cost of disseminating information to farmers and other market players.

## Footnotes

1 It is useful to note that a study by Sheahan and Barrett (2017) of six countries (Nigeria, Niger, Ethiopia, Malawi, Tanzania, and Uganda) found that while average use and adoption levels are extremely low, there is heterogeneity across countries in application and adoption rates. These six countries had application rates higher than the SSA average, partly due to government subsidies. Furthermore, approximately 35% of households use inorganic fertilizer in varying quantities (e.g., 75% in Malawi, 50% in Ethiopia, and around 40% in Nigeria). The study also found that women farmers use relatively less fertilizer than men, the majority of fertilizer purchases by smallholder farmers are made using cash (purchases of fertilizer on credit are insignificant), and small farms use fertilizers more intensively than large farmers. Furthermore, although fertilizer is mainly used on cash crops, noncash staple food crops receive significant inorganic fertilizer use and approximately 50% use of improved seed.
